# Screening of Cytotoxicity and Anti-Inflammatory Properties of Feijoa Extracts Using Genetically Modified Cell Models Targeting TLR2, TLR4 and NOD2 Pathways, and the Implication for Inflammatory Bowel Disease

**DOI:** 10.3390/nu10091188

**Published:** 2018-08-31

**Authors:** Yaoyao Peng, Karen Suzanne Bishop, Lynnette Robin Ferguson, Siew Young Quek

**Affiliations:** 1 Food Science, School of Chemical Sciences, The University of Auckland, Auckland 1010, New Zealand; ypen083@aucklanduni.ac.nz; 2 Discipline of Nutrition and Dietetics, School of Medical Science, Faculty of Medicine and Health Science, The University of Auckland, Auckland 1023, New Zealand; k.bishop@auckland.ac.nz (K.S.B.); l.ferguson@auckland.ac.nz (L.R.F.); 3 Riddet Institute, New Zealand Centre of Research Excellence for Food Research, Palmerston North 4474, New Zealand

**Keywords:** Feijoa fruit extracts, cultivar differences, cytotoxicity, anti-inflammatory pathway, TLR2, NOD2, inflammatory bowel disease

## Abstract

Feijoa has been increasingly studied in the recent decade, while investigations into its bioactivities including anti-inflammatory activity are lacking. In this article, the cytotoxicity and anti-inflammatory properties of feijoa extracts, from flesh, peel and whole fruit, from four cultivars namely APOLLO, UNIQUE, OPAL STAR and WIKI TU are presented. Three inflammatory pathways, Toll-like receptor 2 (TLR2), TLR4 and nucleotide-binding oligomerization domain-containing protein 2 (NOD2), were investigated using genetically modified cell models namely HEK-Blue™ hTLR2, HEK-Blue™ hTLR4, NOD2-WT and NOD2-G908R. Results show that feijoa peel extract induced higher cytotoxicity than flesh and whole fruit extracts, and the APOLLO cultivar was the most anti-inflammatory among the four tested cultivars. The anti-inflammatory activity of feijoa flesh was detected only through the TLR2 pathway, and the activity of feijoa peel and whole fruit was evident mainly through the TLR2 and NOD2 pathways. Most notably, feijoa anti-inflammatory activity was superior to ibuprofen particularly through the TLR2 pathway, with significantly lower secreted embryonic alkaline phosphatase IC50 concentrations (7.88, 12.81, 30.84 and 442.90 μg/mL for APOLLO flesh, peel, whole fruit extract and ibuprofen respectively). These findings indicate that feijoa has great potential to be used in the treatment and prevention of inflammation-related diseases including inflammatory bowel disease.

## 1. Introduction

Feijoa (*Acca sellowiana*) is a subtropical fruit that originated in South America and is now widely grown in New Zealand [1,2]. In the recent decade, the consumption of feijoa has greatly increased and the New Zealand feijoa-processing industries are booming. The health properties of feijoa including antioxidant, antimicrobial and anti-inflammatory activities have been studied [3,4,5,6], but these studies are far from adequate to elucidate the potential benefit of feijoa against a broad range of diseases.

The consumption and processing of feijoa varies depending on different fruit parts. Normally people only consume feijoa flesh, peel is a by-product in feijoa-processing industries (e.g., juicing companies), and the whole fruit is ideal for bioactive compound extraction to be made into natural supplements or medicines. Cultivar differences of a natural plant commonly leads to different chemical compound composition [7,8,9] and, therefore, is an important aspect when considering commercial applications such as functional food development. At present, no clear preference for feijoa cultivar selection is evident from either fresh fruit markets or food-processing industries. The differences in bioactivity among feijoa flesh, peel and whole fruit and different cultivars remain unknown.

One of the traditional applications of feijoas, first investigated by local cultivators from southern Brazil, is for the improvement of intestinal function [10]. The human digestive system is associated with numerous disorders, and one of the most common disorders is chronic inflammation. Drugs such as 5-amino-salicylic acid (5-ASA) are commonly used for the treatment of intestinal inflammation and such drugs inevitably cause side-effects [11]. For this reason, natural methods for treating intestinal inflammatory diseases have received increasing attention. Literature has shown that bioactive compounds from plants, including vegetables and fruits, have potential to alleviate inflammatory symptoms [12,13,14]. A recent study by Nasef et al. [15] has reported feijoa anti-inflammatory activity through the TLR2 pathway. However, more investigations in different pathways are needed to better understand feijoa anti-inflammatory properties. 

Inflammation can be beneficial as it is the auto immune response to harmful stimulations. However, when an acute inflammation develops into chronic inflammation, diseases may result [16]. Pattern recognition receptors (PRRs) are largely involved between pathogenic substances and inflammatory response. PRRs recognize both pathogen-associated molecular patterns (PAMPs) and damage-associated molecular patterns (DAMPs), and pass down the signals for immune responses. Based on the location of the PRRs, they are divided into membrane-bound PRRs including Toll-like receptors (TLRs) and cytoplasmic PRRs including nucleotide-binding oligomerization domain-like receptors, in short NOD-like receptors. The TLR2, TLR4 and NOD2 pathways are among the most widely studied inflammatory pathways [17,18], and participate in many inflammation-related diseases [19]. Furthermore, the NOD2 pathway is specifically related to inflammatory bowel disease (IBD) as a large number of IBD patients harbor NOD2 mutations [20].

The research described herein reports on both the cytotoxicity and anti-inflammatory activity of feijoa extracts, from four feijoa cultivars namely APOLLO, UNIQUE, WIKI TU and OPAL STAR. The four cultivars were carefully selected, with UNIQUE being an early season cultivar; APOLLO being an early to mid season cultivar; WIKI TU being a mid to late season cultivar; and OPAL STAR being a late season cultivar [21,22,23]. These cultivars were all grown in New Zealand and to our knowledge there is no study comparing their bio-functionality. Furthermore, three different sample types, namely flesh, peel and whole fruit, were included in this research, and this is also the first report discussing the differences in terms of cytotoxicity and anti-inflammatory properties among these samples. Three different pathways, TLR2, TLR4 and NOD2, were investigated, focusing on the potential impact of feijoa on inflammatory induced diseases, especially IBD. The objectives of this research were to investigate the potential anti-inflammatory activity and biological pathways acted upon by feijoa fruit, with special emphasis on IBD, and to discuss the differences among feijoa extracts, from flesh, peel and whole fruit, from four cultivars in terms of cytotoxicity and anti-inflammatory properties.

## 2. Materials and Methods

### 2.1. Feijoa Fruits and Extracts

Four feijoa cultivars, namely APOLLO, UNIQUE, WIKI TU and OPAL STAR, were studied in this research. Fresh feijoa fruits were provided from local orchards in the Northern Island of New Zealand during March to May 2017. Feijoa peel and flesh were carefully separated using a manual peeler and freeze dried, along with feijoa whole fruit, immediately after collection. The freeze dried peel, flesh and whole fruit samples, after being ground to powders, were stored at −80 °C until use.

Feijoa extracts were prepared using an optimized ethanol extraction. Briefly, 10 g of freeze-dried feijoa samples were extracted in 500 mL of 50% ethanol at 60 °C for 45 min. The extracted solution was filtered under vacuum and concentrated using rotary evaporation. The final feijoa peel, flesh and whole fruit samples were obtained after freeze-drying. A stock solution of 10 mg/mL for each extract was prepared in dimethyl sulfoxide (DMSO) (Sigma-Aldrich, Corp, St Louis, MO, USA) for cellular tests.

### 2.2. Cell Line and Culture Media

Four mutant cell lines engineered from the Human Embryonic Kidney (HEK) cell line were used in this research. The HEK-Blue™ hTLR2 and HEK-Blue™ hTLR4 cell lines (InvivoGen, San Diego, CA, USA) were made by co-transfection with secreted embryonic alkaline phosphatase (*SEAP*) reporter gene and either the *hTLR2* or the *hTLR4* reporter genes respectively, and therefore they could only be stimulated through the TLR2 and TLR4 receptors. The NOD2-WT and NOD2-G908R cell lines [24,25] were also obtained by co-transfection with a SEAP plasmid, and the NOD2-WT (pUNO-hNOD2a) and G908R single nucleotide polymorphism (SNP) variants respectively. The NOD2-G908R cell line was intended to be less sensitive to ligand stimulation. The four cell lines were designed to study the pathway specific inflammatory effect through NF-κB activation.

The HEK-Blue™ hTLR2 and HEK-Blue™ hTLR4 cell lines were maintained in high glucose Dilbecco’s Modified Eagle’s Medium (DMEM) (Life technologies, Carlsbad, CA, USA) with 10% fetal calf serum (FCS) (Life technologies, Carlsbad, CA, USA), 1% penicillin/streptomycin/glutamine (PSG) (Life technologies, Carlsbad, CA, USA) and 0.4% HEK-Blue™ selection (InvivoGen, San Diego, CA, USA). The NOD2-WT and NOD2-G908R cell lines were similarly maintained in high glucose DMEM with 10% FCS, 1% PSG, and the addition of 0.06% Blasticidin (InvivoGen, San Diego, CA, USA) and 0.1% Zeocin (InvivoGen, San Diego, CA, USA).

### 2.3. Cell Viability Screening of Feijoa Extracts

A sulforhodamine B (SRB) assay was carried out to determine the 50% inhibitory concentrations (IC50) of feijoa extracts on the HEK and NOD2 cell lines. All four cell lines were seeded at a density of 2 × 10^5^ cells/mL in 96 well plates, and incubated in a humidified incubator with 5% CO_2_ at 37 °C for 24 h (Day 1). Feijoa extracts were added to the cell plate from a prepared dosing plate (Day 2). After 48 h, cellular cytotoxicity was measured using the SRB assay [26] (Day 4). Briefly, cells were affixed by adding 40% cold trichloroacetic acid (TCA) (Merck, Darmstadt, Germany), followed by staining using 0.4% SRB dye (Sigma-Aldrich, Corp., St. Louis, MO, USA) in 1% acetic acid (Emsure, Darmstadt, Germany). After the excess dye was washed off with 1% acetic acid in water, 100 μL of 10 mM unbuffered Tris (Invitrogen, Carlsbad, Australia) was added to elute the dye, followed by absorbance measurement at 490 nm wavelength (Bio-Tek Instruments, Winooski, VT, USA).

After the IC50 values were determined, a narrowed concentration range was selected for rapid cytotoxicity screening of all four cultivars on each of the cell lines using the WST-1 assay. Briefly, on Day 4 (following the same seeding and dosing procedures as above), 10 μL of WST-1 (Roche Applied Science, Penzberg, Germany) solution was added into each well and the absorbance was measured at 450 nm (Multiskan, Thermo Labsystems, Waltham, USA) after incubation at 37 °C for 1 h.

### 2.4. Anti-Inflammatory Assay

The same cell-seeding and extract-dosing procedures from Section 2.3 were followed until 48 h after cell seeding. A total of 30 μL of ligand or media, as comparison of with and without ligand stimulation, was added into the plate. The ligands used to stimulate HEK-Blue™ hTLR2 and HEK-Blue™ hTLR4 cell lines were Pam3CysSerLys4 (Pam3CSK4) (InvivoGen, San Diego, CA, USA) and Lipopolysaccharide (LPS) (InvivoGen, San Diego, CA, USA) at a final concentration of 10 ng/mL and 3.125 μg/mL respectively. The ligand for stimulating the NOD2-WT and NOD2-G908R cell lines was Muramyl dipeptide (MDP) (InvivoGen, San Diego, CA, USA) at 22.62 μg/mL. Ibuprofen (Sigma-Aldrich, Corp., St. Louis, MO, USA), Phorbol 12-myristate 13-acetate (PMA) (InvivoGen, San Diego, CA, USA) and 50% DMSO (Sigma-Aldrich, Corp., St. Louis, MO, USA) were also included as the positive, negative and solvent controls respectively. The selected concentration range for Ibuprofen was from 2.14 mM to 0.4 mM (equals to 440.84 μg/mL to 82.40 μg/mL).

After 24 h (Day 4), 30 μL of cell supernatant was removed from each of the 96 wells into a new 96 well plate. A total of 150 μL of QUANTI-Blue (QB) solution (prepared following the manufacturer’s instruction, (InvivoGen, San Diego, CA, USA)) was added into each well and the absorbance was measured at 650 nm (Multiskan, Thermo Labsystems, Waltham, MA, USA) after incubation for 10 min at 37 °C.

### 2.5. Data Analysis

The cell viability values and the SEAP production values were calculated and normalized against cell only control and solvent control respectively. Experiments were conducted in triplicate. Values were expressed as Mean ± SD. The statistical significance was evaluated by one-way analysis of variance (ANOVA) and Tukey B^a,b^ using SPSS 22.0 (IBM Corp., Armonk, NY, USA). Variances were assumed to be homogeneous where *p* > 0.05.

## 3. Results

### 3.1. Feijoa Peel Induced Higher Cytotoxicity than Flesh and Whole Fruit Extracts

To determine the differences in cytotoxicity among the three sample types (flesh, peel and whole fruit extracts) and the four cultivars (APOLLO, UNIQUE, OPAL STAR, WIKI TU), as well as to establish suitable dosing concentrations for the subsequent anti-inflammatory tests, cytotoxicity assays including SRB and WST-1 were conducted. The reason for employing both cytotoxicity assays was because the SRB assay is highly sensitive when cell density is low which makes it accurate in determining IC50 values, while the WST-1 assay is more sensitive in a high cell density environment and suitable for follow-up studies [27].

Firstly, the SRB assay was carried out to determine the IC50 values of APOLLO flesh, peel and whole fruit extracts, on the HEK-Blue™ hTLR2, HEK-Blue™ hTLR4, NOD2-WT and NOD2-G908R cell lines. Results (Table 1) show that the IC50 values of each sample type varied among the four cell lines, and the biggest variation was seen within the flesh extracts (minimum value, 190.5 μg/mL. maximum value, 419.5 μg/mL). Notably, the IC50 values of peel extracts with an average value of 85.08 μg/mL, were much lower than that from the flesh and whole fruit extracts with the average value of 300.88 and 239.28 μg/mL respectively. In other words, peel extract induced higher cytotoxicity in human cells than flesh and whole fruit extracts.

Secondly, a fast cytotoxicity screening of the 12 feijoa extracts (three sample types from four feijoa cultivars) was conducted on the four cell lines using the WST-1 assay. Before commencing the WST-1 screening, several factors for the selection of a suitable concentration range were taken into consideration. Ideally, the highest concentration selected should be lower than the IC50 from the SRB assay so that the extracts are not toxic. The concentration range should be consistent across all sample types so that the results from all samples are comparable. However, due to the significantly lower SRB IC50 value from the peel extract (Table 1), we chose a concentration around 70 μg/mL for the peel extracts, and a 2-fold higher concentration for the flesh and the whole fruit extracts. After some optimizations, the concentrations tested were finally determined as 142.86 μg/mL, 71.34 μg/mL, 35.71 μg/mL, 17.86 μg/mL, 8.93 μg/mL and 4.46 μg/mL.

The results of WST-1 screening are presented in Table 2. Generally, the cell viability decreased when the extract concentrations increased, and no significant differences (*p* > 0.05) were observed at relatively low concentrations. For example, as seen with the treatment of APOLLO whole fruit extract on the HEK-Blue™ hTLR2 cell line, the 142.86 μg/mL concentration had a significantly lower cell viability (0.66 ± 0.07) than the rest of the concentrations, while concentrations from 71.34 μg/mL to 8.93 μg/mL showed no significant difference among each other although they all had significantly lower cell viability than the 4.46 μg/mL concentration. Summarized from the WST-1 results (Table 2), within the selected concentration range, the flesh and the whole fruit extracts from all cultivars induced minor toxicity (average cell viability above 0.6) in the HEK-Blue and NOD2 cell lines at the highest concentration tested (142.86 μg/mL), and negligible toxicity (average cell viability above 0.8) at a concentration of 71.34 μg/mL. While the peel extracts, consistent with the results from the SRB assay, induced lower cell viability than the flesh and the whole fruit extracts. Such differences among the three sample types were particularly significant in the APOLLO and UNIQUE cultivars (Table 2).

Cultivar difference was not significant except that the peel extracts from OPAL STAR and WIKI TU cultivar had higher cell viability than the APOLLO and UNIQUE cultivars on the HEK-Blue™ hTLR2 and HEK-Blue™ hTLR4 cell lines at concentrations of 142.86 and 71.34 μg/mL (Table 2).

### 3.2. Potential Anti-Inflammatory Activity of Feijoa through TLR2 and TLR4 Pathways

The HEK-Blue ™ hTLR2 and HEK-Blue™ hTLR4 cell line are genetically modified cell lines that can only be stimulated through the TLR2 and TLR4 pathways, making them good cell models to investigate anti-inflammatory pathways. A QUANTI-Blue assay was applied to test anti-inflammatory activity by detecting the level of SEAP from cell supernatant, and a low SEAP secretion (relative to control) indicates a strong anti-inflammatory effect. As peel extracts at the concentration of 142.86 μg/mL were shown to have a moderate cytotoxicity effect on the HEK-Blue cells, with the cell viability (less than 0.6) significantly less than that measured when lower concentrations were used (Table 2), therefore the highest concentration for the treatment with peel extracts was chosen at 71.34 μg/mL.

**Figure 1:**
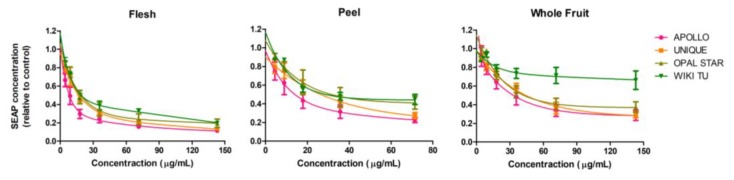
Anti-inflammatory activity in the HEK-Blue™ hTLR2 cell line. Cells were treated with feijoa flesh, peel and whole fruit extracts from APOLLO, UNIQUE, OPAL STAR and WIKI TU cultivars. PAM3CSK4 ligand was used to stimulate an inflammatory response through the TLR2 pathway. Secreted embryonic alkaline phosphatase (SEAP) concentration was normalized against solvent control. A low SEAP concentration indicates a strong anti-inflammatory effect.

As shown in Figure 1, all the extracts strongly suppressed SEAP production in the HEK-Blue™ hTLR2 cell line in a dose-dependent manner. Generally, the SEAP expression of HEK-Blue™ hTLR2 cell line treated with feijoa extracts decreased sharply when the extract concentrations increased from 4.46 to 35.71 μg/mL, and gradually became stable from 35.71 to 142.86 μg/mL. Cultivar variation can be seen slightly from the flesh and the peel extracts, but significantly from the whole fruit extract with WIKI TU cultivar being less effective than the other cultivars.

To better evaluate and compare the anti-inflammatory activity of feijoa extracts through the TLR2 pathway regarding the different sample types and cultivars, the inhibition of SEAP expression at 30%, 50% and 70% (IC30, IC50, IC70) in the HEK-Blue™ hTLR2 cell line was calculated (Table 3) according to the best-fit curve within the selected concentration range. The data from the positive control, ibuprofen, was also presented. Results show that the following extracts achieved IC70 SEAP expression. They are flesh extracts from all cultivars, peel extracts from the APOLLO and UNIQUE cultivars, and whole fruit extract only from the APOLLO cultivar. Among the three sample types, flesh extracts showed the strongest anti-inflammatory activity with a lower concentration in reaching IC30, IC50 and IC70 SEAP expression, followed by the peel extract and the whole fruit extract. In terms of cultivars, APOLLO generated the most effective anti-inflammatory extracts as all extracts achieved IC70 SEAP expression, while the WIKI TU cultivar was the least anti-inflammatory. In addition, the anti-inflammatory efficiency of feijoa extracts through the TLR2 pathway was competitive, even superior, to the well-known anti-inflammatory drug ibuprofen regarding the significantly lower SEAP IC50 concentrations and the achievement of SEAP IC70 (Table 3).

**Figure 2:**
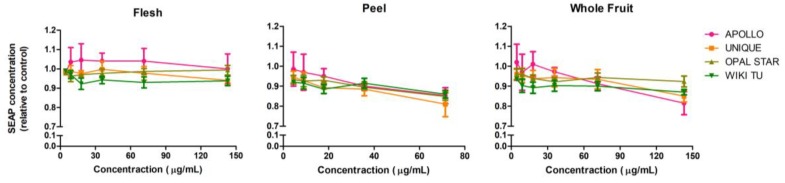
Anti-inflammatory activity on the HEK-Blue™ hTLR4 cell line. Cells were treated with feijoa flesh, peel and whole fruit extracts from APOLLO, UNIQUE, OPAL STAR and WIKI TU cultivars. Lipopolysaccharide (LPS) ligand was used to stimulate an inflammatory response through the TLR4 pathway. The SEAP concentration was normalized against the solvent control. A low SEAP concentration indicates a strong anti-inflammatory effect.

As shown in Figure 2, feijoa extracts had very limited anti-inflammatory effects on the HEK-Blue™ hTLR4 cell line. The flesh extracts from all four cultivars showed no effect in reducing SEAP production, while very slight decreasing trends were observed from peel and whole fruit extracts. Therefore, feijoa flesh extract specifically mediates the TLR2 anti-inflammatory pathway rather than TLR4, while the effects of peel and whole fruit extracts are mainly through TLR2 and to a much lesser extent through TLR4 pathway.

### 3.3. Activation of the Nucleotide-Binding Oligomerization Domain-Containing Protein 2 (NOD2) Anti-Inflammation Pathway

Based on the screening results of feijoa anti-inflammatory activity on the HEK-Blue™ hTLR2 and HEK-Blue™ hTLR4 cell lines, the APOLLO cultivar was chosen as a representative to further study the potential anti-inflammatory activity of feijoa through the NOD2 pathway. A series concentration from 142.86 to 4.46 μg/mL was employed to treat the NOD2 cells based on the cell viability screening results (Table 2).

**Figure 3:**
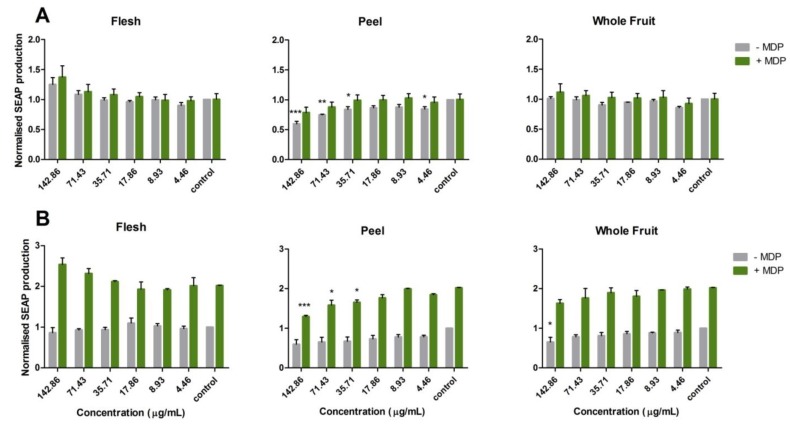
Anti-inflammatory activity of feijoa extracts on the (**A**) NOD2-G908R and (**B**) NOD2-WT cell lines. Cells were treated with feijoa flesh, peel and whole fruit extracts from APOLLO cultivars. Muramyl dipeptide (MDP) ligand was used to stimulate an inflammatory response through the NOD2 pathway. A normalized SEAP production lower than in the control indicates a potential anti-inflammatory effect. Statistical analysis was carried out using one-way analysis of variance (ANOVA) (*** *p* < 0.001, ** *p* < 0.01, * *p* < 0.05).

As shown in Figure 3, the NOD2-G908R cell line was far less sensitive to MDP stimulation than the NOD2-WT cell line as expected. The NOD2-WT cell line, after ligand stimulation, had approximately 2-fold higher expression of SEAP than that seen without ligand stimulation. However, no significant increase of SEAP expression was observed in the NOD2-G908R cell line with ligand stimulation. 

It is interesting to note that the feijoa flesh, peel and whole fruit extracts (APOLLO cultivar) did not show the same pattern of effect on the SEAP expression induced by MDP stimulation. Flesh extract showed an increasing trend as the dosing concentration increased from 4.46 μg/mL to 142.86 μg/mL, particularly when the cells were stimulated with MDP, but no significant difference was observed relative to the control. In other words, feijoa flesh extracts had no impact on the reduction of SEAP expression. As for the whole fruit extract, a significant SEAP suppression was detected at 142.86 μg/mL compared to the control on the NOD2-WT cell line without ligand stimulation, and there was no significant trend observed when stimulated by ligand. On the other hand, no significant impact can be seen either with or without ligand stimulation on the NOD2-G908R cell line. Nevertheless, peel extract showed a significant suppressive effect of SEAP production on both NOD2-WT and NOD2-G908R cell lines. Significant results were observed on the NOD2-G908R cell line without ligand stimulation at the concentrations of 142.86 (*p* < 0.001), 71.43 (*p* < 0.01), 35.71 (*p* < 0.05) and 4.46 (*p* < 0.05) μg/mL, and on the NOD2-WT cell line with ligand stimulation at the concentrations of 142.86 (*p* < 0.001), 71.43 (*p* < 0.05) and 35.71 (*p* < 0.05) μg/mL (Figure 3).

As a summary for the NOD2 pathway, feijoa flesh extract had no anti-inflammatory impact, the whole fruit extract has some potential anti-inflammatory effect, while the peel extract significantly suppressed inflammation (*p* < 0.001).

## 4. Discussion

Feijoa flesh, peel and whole fruit samples were extracted using feijoa inner pulp, outer green layer and intact fruit respectively. Due to the different applications of the three sample types, it is worth investigating the differences among them with respect to cytotoxicity and anti-inflammatory properties. Cultivar difference is also important for feijoa functional food development. Different feijoa cultivars may show obvious phenotypic differences, in terms of fruit shape, size, color, texture and taste, due to various genotypes or/and cultivation conditions [1]; it is possible that different cultivars possess different functional aspects or/and strength. Since no existing study has suggested feijoa cultivar selection for functional food development, our study is essential to provide scientific evidence on whether different cultivars significantly vary with regard to their bioactivities.

Determining the cytotoxicity level of an extract is essential and fundamental to investigating its potential bioactivities. In this research, two methods, SRB and WST-1 assays, were included to investigate feijoa cytotoxicity levels. The theory behind the SRB assay is using SRB dye to bind cellular proteins, and in this way the amount of dye, once eluted from the stained cells, is in proportion to the cell numbers [26]. This method is frequently used in IC50 determination due to its stable reaction, and the IC50 value is widely used as the assessment of cytotoxicity from extracts or compounds [28,29].

WST-1 is a relative new method used in the quantification of cell proliferation, cell viability and cytotoxicity [24,30]. Its working principle is to allow the cellular enzymes secreted by active cells to cleave the tetrazolium salts into formazan, which means the amount of formazan is correlated to the number of active cells [31]. In other words, the SRB assay detected both viable and dead cells while the WST-1 assay detected only viable cells. Therefore, it is critical for our study to include the WST-1 assay, as the follow-up anti-inflammatory tests were only reliable when the treated cells were alive and viable. Furthermore, compared to the complexity of multiple washing and drying steps that have to be performed for the SRB assay, the biggest advantage of the WST-1 assay is one-step reagent addition and fast reaction time, and hence the WST-1 assay is ideal as a rapid, high throughput assay for the comparison of large samples numbers [32]. Therefore, in this research, the SRB assay was carried out to initially establish an IC50 value for each sample type using the APOLLO cultivar, while WST-1 assay was conducted as a rapid comparison as well as double-confirmation of cytotoxicity level of feijoa extracts from all four cultivars using the already narrowed concentration range.

The HEK-Blue™ hTLR2, HEK-Blue™ hTLR4 and NOD2 cell lines in this research are genetically modified cell lines that can only be stimulated through TLR2, TLR4 and NOD2 pathways respectively. All three pathways are associated with IBD [33]. The TLR2 and TLR4 pathways are mediated by the Toll-like 2 and 4 receptors belonging to the membrane-bound PRRs, while the NOD2 pathway is regulated by the NOD2 receptor belonging to the cytoplasmic PRRs. By examining the three anti-inflammatory pathways, we were able to assess whether feijoa extracts have anti-inflammatory effects, whether different feijoa samples or cultivars have different anti-inflammatory strength and/or different mechanisms of action, and whether feijoa could potentially be used against inflammatory induced diseases, for example IBD.

According to our results (Table 1 and Table 2), peel extract induced significantly lower cell viability than the flesh and whole fruit samples, indicating that peel extract, as a potential by-product for functional foods, more rapidly causes toxic effects than the flesh and the whole fruit extracts in human cell lines. The reason could lie in the compounds found in feijoa peel, which is a natural protection against insects and bacteria. One example of protection afforded by peel was presented by Salvatore et al. [34] who demonstrated that lemon peel was toxic to the fruit fly *Ceratitis capitata*. Feijoa flesh and whole fruit extracts were mostly not significantly different regarding the cell viability of the tested cell lines, indicating that as long as feijoa peel is not consumed alone in large quantities (e.g., use as food additives), the potential cytotoxic compounds in peel do not affect the suitability of whole feijoa as an edible fruit. 

All tested feijoa extracts exhibited significant anti-inflammatory activity, particularly through the TLR2 pathway, which is in agreement with a previous study on feijoa anti-inflammatory activity [15]. However, no significant anti-inflammatory effects were seen with the peel and whole fruit extracts through the TLR4 pathway, indicating that the anti-inflammatory activity of feijoa extract might be pathway specific. Among the three sample types, feijoa flesh extracts showed the strongest anti-inflammatory activity through the TLR2 pathway (Table 3), which is somewhat surprising because peel was frequently found to be more bioactive than flesh in other fruits [35,36]. This requires further investigation in the identification of bioactive compounds in feijoa flesh and peel. Furthermore, our results suggest that the APOLLO cultivar was the best among the four tested cultivars regarding anti-inflammatory activity through the TLR2 pathway. Moreover, the quantified anti-inflammatory strength of feijoa through the TLR2 pathway was superior to the well-known drug ibuprofen, indicating feijoa’s great potential to be developed into natural medicines. 

As for the NOD2 pathway, the comparison of the two NOD2 cell lines could help to understand the activation of the NOD2 reporter in the regulation of the NOD2 anti-inflammatory pathway as the NOD2-G908R cell line was designed to be less sensitive than the WT to MDP stimulation. Moreover, the G908R SNP (Gly908Arg) is one of the most common SNPs in NOD2 that is associated with Crohn’s disease [37]. Crohn’s disease is one of the most common types of IBD, which makes the NOD2-G908R cell line a useful model to investigate potential functional foods to alleviate symptoms of IBD. Flesh extracts, at relatively high concentrations, increased SEAP expression, suggesting a potential pro-inflammatory effect. The whole fruit extract showed a slight decreasing trend on SEAP expression for both with and without ligand stimulation on the NOD2-WT cell line, indicating a potential weak anti-inflammatory effect through the NOD2 pathway and the effect targeted the NOD2 reporter itself. This requires further experimentation, such as gene expression, to confirm the mechanism of action. Peel extracts showed clear anti-inflammatory activity through the NOD2 pathway. However, with the observation of the anti-inflammatory effect on both NOD2 cell lines, the mechanism of the activation of the NOD2 pathway by feijoa peel extracts could be the regulation of the downstream signaling molecules of the NOD2 pathway. A similar finding was presented by Folkard et al. [24] in their study of sulforaphane. The successful application of the NOD2 cell models strongly suggested that feijoa peel extracts could be developed into anti-IBD supplements or medicines.

As commonly understood, the anti-inflammatory activity of natural plants originates from the phytochemicals including, but not limited to, phenolic compounds [38], monoterpenes [39] and phytosterols [40]. It is important to identify the bioactive compounds of feijoa for potential medical use. However, at present, there are still very limited studies focusing on the characterization of feijoa bioactive compounds, and the dominant anti-inflammatory compounds in feijoa remain unknown. Some research has stated that feijoa is rich in phenolic compounds [41,42], and the phenolic compounds could play an important role in the anti-inflammatory activity of feijoa. Aoyama, Sakagami and Hatano [43] identified seven phenolic compounds including flavone and ellagic acid in feijoa fruit, although their study did not involve anti-inflammatory tests, this information could be useful for future studies on feijoa bioactivities, including anti-inflammatory activity.

In summary, feijoa flesh, peel and whole fruit extracts all showed strong anti-inflammatory activity although they might act through one or more different pathways, and the four different feijoa cultivars had the same anti-inflammatory patterns but with different anti-inflammatory potency. This may have resulted due to the variation in type and quantity of bioactive compounds in different feijoa samples and cultivars. Anti-inflammatory pathways of feijoa extracts, particular from peel samples, could work synergistically to stimulate inflammatory mediators. This hypothesis was supported by other studies [44,45]. In terms of IBD, since no universal cure is available, personalized dietary intervention using fruits and vegetables is highly recommended in both the treatment and the prevention of IBD. This research demonstrated that feijoa has the potential to be developed into functional foods and natural medicine to assist controlling inflammatory-induced diseases such as IBD.

## 5. Conclusions

Feijoa peel extracts induced higher cytotoxicity than the flesh and whole fruit extracts. Among the four cultivars assessed, APOLLO could be the most promising source to be utilized to reduce inflammation. Significant anti-inflammatory activity was detected through the TLR2 pathway for all sample types, and the NOD2 pathway for peel and whole fruit samples, while no significant effect was seen in the TLR4 pathway. Remarkably, the anti-inflammatory activity of feijoa through the TLR2 pathway was superior to ibuprofen. These findings indicated that feijoa has a great potential to be developed into functional foods, natural supplements or medicines against inflammatory related diseases including IBD. The application of feijoa extracts from different sample types could vary due to the nature of the fruit. Feijoa flesh, as the major edible part of the fruit, could assist in the prevention and regulation of inflammatory related disease when used as a dietary intervention. Feijoa peel, as the by-product of feijoa-processing industries, could be reused in functional foods or natural medicines. Feijoa whole fruit, either eaten fresh or used in the development of natural supplements, could significantly contribute to the maintenance of human health and treatment of disease.

## Figures and Tables

**Table 1:** IC50 Cytotoxicity of APOLLO extracts on the HEK-Blue and nucleotide-binding oligomerization domain-containing protein 2 (NOD2) cell lines using the sulforhodamine B (SRB) assay.

Cell Line	Flesh (μg/mL)	Peel (μg/mL)	Whole Fruit (μg/mL)
HEK-Blue™ hTLR2	419.50	77.19	252.80
HEK-Blue™ hTLR4	360.60	79.17	257.20
NOD2-WT	190.50	83.57	200.00
NOD2-G908R	232.90	100.40	247.10
Mean (SD)	300.88 ± 92.79	85.08 ± 9.14	239.28 ± 22.96

**Table 2:** Cell viability of the HEK-Blue and NOD2 cell lines treated with feijoa extracts (WST-1 assay).

Conc. (μg/mL)	HEK-Blue™ hTLR2			HEK-Blue™ hTLR4			NOD2-WT			NOD2-G908R		
	F	P	WF	F	P	WF	F	P	WF	F	P	WF
**APOLLLO**												
142.86	0.63 ± 0.06 ^ab/BC^ *	0.54 ± 0.09 ^a/AB^ *	0.66 ± 0.07 ^a/BC^ *	0.66 ± 0.10 ^ab/BC^	0.41 ± 0.07 ^a/A^ *	0.64 ± 0.05 ^a/BC^ *	0.72 ± 0.13 ^ab/BC^	0.57 ± 0.12 ^abc/BC^	0.69 ± 0.13 ^ab/BC^	0.71 ± 0.04 ^ab/BC^	0.63 ± 0.05 ^abc/BC^	0.73 ± 0.08 ^abc/C^
71.43	0.80 ± 0.05 ^cdef/BC^	0.60 ± 0.06 ^a/A^ *	0.83 ± 0.04 ^bcd/BC^	0.78 ± 0.07 ^bcdef/BC^	0.57 ± 0.07 ^b/A^ *	0.80 ± 0.05 ^cde/BC^	0.88 ± 0.06 ^ab/C^	0.68 ± 0.12 ^abcde/AB^	0.85 ± 0.14 ^ab/BC^	0.79 ± 0.04 ^abcd/BC^	0.76 ± 0.12 ^abcdefg/BC^	0.88 ± 0.16 ^abcde/BC^
35.71	0.86 ± 0.08 ^defg/A^	0.86 ± 0.04 ^cde/A^	0.90 ± 0.02 ^de/A^	0.81 ± 0.06 ^cdefg/A^	0.78 ± 0.08 ^defg/A^	0.87 ± 0.04 ^efgh/A^	0.94 ± 0.10 ^ab/A^	0.78 ± 0.08 ^bcdef/A^	0.94 ± 0.11 ^b/A^	0.87 ± 0.06 ^bcd/A^	0.85 ± 0.08 ^cdefghi/A^	0.91 ± 0.10 ^abcde/A^
17.86	0.90 ± 0.06 ^efg/A^	0.91 ± 0.03 ^de/A^	0.92 ± 0.03 ^de/A^	0.88 ± 0.05 ^fgh/A^	0.87 ± 0.08 ^efgh/A^	0.90 ± 0.06 ^efgh/A^	0.96 ± 0.10 ^ab/A^	0.89 ± 0.12 ^def/A^	1.00 ± 0.15 ^b/A^	0.89 ± 0.09 ^bcd/A^	1.00 ± 0.23 ^defghi/A^	1.04 ± 0.24 ^abcde/A^
8.93	0.92 ± 0.06 ^fg/A^	0.93 ± 0.04 ^de/A^	0.96 ± 0.03 ^de/A^	0.93 ± 0.06 ^gh/A^	0.92 ± 0.06 ^hi/A^	0.94 ± 0.04 ^gh/A^	1.01 ± 0.11 ^ab/A^	0.91 ± 0.08 ^ef/A^	1.02 ± 0.12 ^b/A^	0.91 ± 0.09 ^bcd/A^	0.94 ± 0.09 ^fghi/A^	1.00 ± 0.14 ^cde/A^
4.46	0.94 ± 0.11 ^g/A^	0.96 ± 0.03 ^e/A^	0.97 ± 0.04 ^e/A^	0.98 ± 0.08 ^h/A^	0.99 ± 0.04 ^i/A^	0.98 ± 0.04 ^h/A^	1.04 ± 0.11 ^b/A^	0.96 ± 0.10 ^ef/A^	1.07 ± 0.15 ^b/A^	0.94 ± 0.08 ^d/A^	0.98 ± 0.08 ^ghi/A^	1.02 ± 0.11 ^de/A^
**UNIQUE**												
142.86	0.63 ± 0.08 ^ab/BCD^ *	0.57 ± 0.07 ^a/ABC^ *	0.71 ± 0.06 ^a/CD^ *	0.72 ± 0.08 ^abcde/CD^	0.51 ± 0.06 ^a/A^ *	0.65 ± 0.06 ^ab/BCD^ *	0.69 ± 0.15 ^a/CD^	0.44 ± 0.12 ^a/A^	0.50 ± 0.12 ^a/AB^	0.77 ± 0.05 ^abcd/D^	0.56 ± 0.04 ^a/ABC^	0.65 ± 0.04 ^a/BCD^
71.43	0.79 ± 0.06 ^cde/CD^	0.74 ± 0.04 ^b/BCD^ *	0.83 ± 0.04 ^bcd/D^	0.82 ± 0.09 ^cdefg/D^	0.60 ± 0.09 ^bc/AB^ *	0.80 ± 0.06 ^cde/CD^	0.79 ± 0.11 ^ab/CD^	0.53 ± 0.14 ^ab/A^	0.66 ± 0.07 ^ab/BC^	0.83 ± 0.05 ^abcd/D^	0.70 ± 0.04 ^abcde/BCD^	0.81 ± 0.04 ^abcde/CD^
35.71	0.83 ± 0.03 ^cdefg/ABCD^	0.88 ± 0.03 ^cde/BCD^	0.86 ± 0.03 ^bcde/BCD^	0.87 ± 0.10 ^fgh/BCD^	0.77 ± 0.06 ^def/ABC^	0.84 ± 0.07 ^defg/BCD^	0.80 ± 0.07 ^ab/ABCD^	0.69 ± 0.10 ^abcde/A^	0.75 ± 0.07 ^ab/AB^	0.91 ± 0.06 ^bcd/CD^	0.92 ± 0.07 ^defghi/D^	0.92 ± 0.08 ^bcde/D^
17.86	0.85 ± 0.06 ^cdefg/AB^	0.90 ± 0.03 ^de/AB^	0.89 ± 0.06 ^cde/AB^	0.90 ± 0.08 ^fgh/AB^	0.85 ± 0.04 ^efgh/AB^	0.89 ± 0.09 ^efgh/AB^	0.87 ± 0.15 ^ab/AB^	0.89 ± 0.08 ^ef/AB^	0.91 ± 0.24 ^b/A^	0.89 ± 0.06 ^bcd/AB^	1.00 ± 0.07 ^hi/B^	0.98 ± 0.09 ^cde/B^
8.93	0.89 ± 0.05 ^efg/AB^	0.92 ± 0.06 ^de/AB^	0.93 ± 0.04 ^de/AB^	0.89 ± 0.09 ^gh/AB^	0.90 ± 0.04 ^ghi/AB^	0.90 ± 0.07 ^efgh/AB^	0.82 ± 0.06 ^ab/A^	0.94 ± 0.11 ^ef/AB^	0.93 ± 0.17 ^b/A^	0.86 ± 0.07 ^bcd/AB^	1.01 ± 0.10 ^i/B^	1.01 ± 0.07 ^cde/B^
4.46	0.92 ± 0.05 ^fg/ABCD^	0.94 ± 0.04 ^de/ABCD^	0.95 ± 0.05 ^de/BCD^	0.90 ± 0.04 ^h/ABC^	0.91 ± 0.03 ^hi/ABCD^	0.92 ± 0.04 ^fgh/ABCD^	0.82 ± 0.05 ^ab/A^	1.02 ± 0.10 ^f/CD^	0.93 ± 0.16 ^b/AB^	0.84 ± 0.03 ^bcd/AB^	1.03 ± 0.08 ^hi/D^	1.00 ± 0.05 ^cde/CD^
**OPAL STAR**												
142.86	0.62 ± 0.04 ^a/ABC^	0.58 ± 0.07 ^a/AB^ *	0.70 ± 0.05 ^a/ABCD^	0.68 ± 0.07 ^abc/ABCD^	0.59 ± 0.08 ^bc/AB^	0.72 ± 0.08 ^abc/BCD^	0.69 ± 0.06 ^a/ABCD^	0.56 ± 0.08 ^abc/A^	0.66 ± 0.04 ^ab/ABCD^	0.77 ± 0.02 ^abcd/CD^	0.66 ± 0.06 ^abcd/ABCD^	0.78 ± 0.06 ^abcd/D^
71.43	0.73 ± 0.03 ^abc/AB^	0.78 ± 0.05 ^bc/AB^	0.76 ± 0.05 ^abc/AB^	0.71 ± 0.06 ^abcde/AB^	0.69 ± 0.07 ^cd/A^	0.79 ± 0.06 ^cde/AB^	0.78 ± 0.01 ^ab/AB^	0.68 ± 0.08 ^abcde/A^	0.79 ± 0.00 ^ab/AB^	0.85 ± 0.07 ^abcd/B^	0.80 ± 0.10 ^bcdefgh/AB^	0.86 ± 0.04 ^abcde/B^
35.71	0.83 ± 0.06 ^cdefg/AB^	0.86 ± 0.06 ^cde/AB^	0.84 ± 0.08 ^bcde/AB^	0.79 ± 0.05 ^bcdefg/A^	0.83 ± 0.08 ^efgh/AB^	0.86 ± 0.06 ^defg/AB^	0.84 ± 0.02 ^ab/AB^	0.79 ± 0.06 ^bcdef/A^	0.86 ± 0.04 ^ab/AB^	0.88 ± 0.04 ^bcd/AB^	0.91 ± 0.07 ^efghi/AB^	0.96 ± 0.10 ^bcde/B^
17.86	0.86 ± 0.04 ^defg/AB^	0.89 ± 0.04 ^cde/AB^	0.90 ± 0.08 ^de/AB^	0.81 ± 0.05 ^cdefg/A^	0.87 ± 0.08 ^efgh/AB^	0.85 ± 0.09 ^defg/AB^	0.90 ± 0.05 ^ab/AB^	0.89 ± 0.03 ^def/AB^	0.93 ± 0.06 ^b/AB^	0.89 ± 0.04 ^bcd/AB^	0.98 ± 0.08 ^ghi/B^	0.98 ± 0.05 ^cde/B^
8.93	0.88 ± 0.04 ^efg/A^	0.92 ± 0.06 ^de/A^	0.91 ± 0.05 ^de/A^	0.82 ± 0.06 ^cdefg/A^	0.87 ± 0.07 ^efgh/A^	0.88 ± 0.06 ^efgh/A^	0.91 ± 0.06 ^ab/A^	0.94 ± 0.06 ^ef/AB^	0.98 ± 0.12 ^b/A^	0.93 ± 0.05 ^cd/AB^	0.97 ± 0.10 ^ghi/AB^	1.08 ± 0.07 ^e/B^
4.46	0.88 ± 0.06 ^efg/AB^	0.94 ± 0.04 ^de/AB^	0.97 ± 0.05 ^e/AB^	0.83 ± 0.08 ^defg/A^	0.88 ± 0.11 ^fghi/AB^	0.91 ± 0.08 ^efgh/AB^	0.93 ± 0.08 ^ab/AB^	0.98 ± 0.04 ^ef/AB^	1.01 ± 0.07 ^b/AB^	0.90 ± 0.03 ^bcd/B^	0.99 ± 0.10 ^ghi/AB^	1.03 ± 0.02 ^de/AB^
**WIKI TU**												
142.86	0.74 ± 0.08 ^ab/BC^ *	0.58 ± 0.08 ^a/A^ *	0.76 ± 0.09 ^ab/C^	0.64 ± 0.04 ^a/ABC^	0.60 ± 0.06 ^bc/AB^ *	0.67 ± 0.04 ^ab/ABC^	0.68 ± 0.05 ^a/ABC^	0.60 ± 0.06 ^abcd/AB^	0.72 ± 0.07 ^ab/ABC^	0.65 ± 0.03 ^a/ABC^	0.60 ± 0.03 ^ab/AB^	0.68 ± 0.02 ^ab/ABC^
71.43	0.85 ± 0.09 ^cdefg/A^	0.82 ± 0.12 ^bcd/A^	0.87 ± 0.10 ^bcde/A^	0.70 ± 0.04 ^abcd/A^	0.75 ± 0.06 ^de/A^	0.74 ± 0.04 ^bcd/A^	0.81 ± 0.08 ^ab/A^	0.72 ± 0.07 ^bcde/A^	0.84 ± 0.10 ^ab/A^	0.71 ± 0.04 ^ab/A^	0.72 ± 0.04 ^abcdef/A^	0.79 ± 0.04 ^abcde/A^
35.71	0.86 ± 0.10 ^defg/A^	0.86 ± 0.07 ^cde/A^	0.90 ± 0.10 ^de/A^	0.77 ± 0.07 ^abcdef/A^	0.82 ± 0.05 ^efgh/A^	0.79 ± 0.04 ^cde/A^	0.79 ± 0.05 ^ab/A^	0.81 ± 0.07 ^cdef/A^	0.85 ± 0.08 ^ab/A^	0.73 ± 0.06 ^abc/A^	0.81 ± 0.02 ^abcdefg/A^	0.81 ± 0.04 ^abcde/A^
17.86	0.91 ± 0.10 ^fg/AB^	0.89 ± 0.09 ^cde/AB^	0.93 ± 0.11 ^de/B^	0.78 ± 0.06 ^bcdef/AB^	0.84 ± 0.03 ^efgh/AB^	0.82 ± 0.06 ^cdef/AB^	0.85 ± 0.09 ^ab/AB^	0.88 ± 0.06 ^def/AB^	0.93 ± 0.07 ^b/B^	0.73 ± 0.06 ^abcd/A^	0.88 ± 0.03 ^defghi/AB^	0.81 ± 0.08 ^abcde/AB^
8.93	0.93 ± 0.09 ^fg/AB^	0.92 ± 0.08 ^de/AB^	0.97 ± 0.10 ^e/B^	0.86 ± 0.08 ^efgh/AB^	0.86 ± 0.03 ^efgh/AB^	0.84 ± 0.04 ^defg/AB^	0.91 ± 0.13 ^ab/AB^	0.89 ± 0.06 ^def/AB^	0.95 ± 0.04 ^b/B^	0.74 ± 0.09 ^abcd/A^	0.89 ± 0.04 ^defghi/AB^	0.84 ± 0.04 ^abcde/AB^
4.46	0.95 ± 0.09 ^g/AB^	0.94 ± 0.06 ^de/AB^	0.97 ± 0.06 ^e/AB^	0.90 ± 0.03 ^fgh/AB^	0.91 ± 0.02 ^hi/AB^	0.90 ± 0.03 ^efgh/AB^	0.87 ± 0.13 ^ab/AB^	0.97 ± 0.06 ^ef/AB^	1.02 ± 0.12 ^b/B^	0.79 ± 0.07 ^abcd/A^	0.82 ± 0.07 ^bcdefghi/AB^	0.87 ± 0.08 ^abcde/AB^

(Mean ± SD, F—flesh extracts, P—peel extracts, WF—whole fruit extracts, *n* ≥ 3; ^abcdefghi^, for significance comparison by each column; ^ABCD^, for significance comparison by each row); Statistical analysis was carried out using Tukey B^a,b^; Extract concentrations that induced significantly lower cell viability within the concentration range were highlighted in bold and marked with *.

**Table 3:** Inhibition of SEAP expression by feijoa extracts and ibuprofen on the HEK-Blue™ hTLR2 cell line.

	APOLLO (μg/mL)			UNIQUE (μg/mL)			OPAL STAR (μg/mL)			WIKI TU (μg/mL)			IB (μg/mL)
	F	P	WF	F	P	WF	F	P	WF	F	P	WF	
**IC30**	4.06	5.87	11.90	7.26	9.31	18.35	8.04	11.12	16.32	8.37	11.86	89.38	230.72
**IC50**	7.88	12.81	30.84	13.70	26.68	55.80	16.70	31.74	48.40	17.97	41.24	N/A	442.90
**IC70**	21.42	42.32	133.31	35.90	76.45	N/A	50.67	N/A	N/A	57.26	N/A	N/A	N/A

(F—flesh extracts, P—peel extracts, WF—whole fruit extracts, IB—ibuprofen; N/A—not achieved within selected concentrations).
